# Mental Health in the Millennium Development Goals: Not Ignored

**DOI:** 10.1371/journal.pmed.0040056

**Published:** 2007-01-30

**Authors:** Sonia Ehrlich Sachs, Jeffrey D Sachs

In a *PloS Medicine* article of September 2005, J. Jaime Miranda and Vikram Patel ask: “Achieving the Millennium Development Goals [MDGs]: Does Mental Health Play a Role?” [1]. We agree with their concern that “there is no health without mental health.” However, we do not feel mental health is ignored in the health agenda, nor do we share their pessimism about the potential to reach the MDGs in general.

Skepticism about the success of the MDGs is based on the poor track record of past international goals such as the Universal Declaration of Human Rights or the Declaration of Alma-Ata. Indeed, the MDGs were adopted with these pitfalls in mind. Emphasis was given to setting bold but realistic goals, with quantifiable, time-bound targets. For example, the aim to “reduce by two-thirds, between 1990 and 2015, the under-five mortality rate,” calls for a practical plan with concrete, monitorable guideposts.

Many assessments have shown how these health goals can be achieved over the next ten years. The fact that progress on under-5 mortality and disease control has been too slow and that previous goals have not been met is why the world needs the MDGs. Without these targets that hold poor and rich countries accountable, poor countries will miss the benchmarks laid out in the Millennium Declaration, even though the objectives are attainable.

The reason that the MDGs do not explicitly address noncommunicable diseases such as cardiovascular or psychiatric diseases is that the MDGs focus on the gap in health status between rich and poor countries, a gap mainly accounted for by infectious diseases, malnutrition, and unsafe childbirth. The goals were crafted to address these large gaps rather than to solve all pressing health problems.

We agree that “mental illness is closely associated with social determinants, notably poverty and gender disadvantage, and with poor physical health, including having HIV/AIDS and poor maternal and child health.” About 2.6% of disability-adjusted life years in sub-Saharan Africa are attributable to psychiatric conditions, which is about the same proportion attributable to nutritional deficiencies, or tuberculosis, or maternal complications from childbirth [2]. There are many areas in which governments are using MDG-based strategies to tackle problems that are not explicitly mentioned by the MDGs, such as electrification, road construction, increased agricultural yields, and more.

The author's contention that “the MDGs do not address strengthening of health systems” is not correct, as readers of the UN Millennium Project recommendations (http://www.unmillenniumproject.org) can see. There is no chance to achieve any health MDGs without strengthening the health systems. Low-income countries are placing a important emphasis on strengthening health systems in their MDG-based planning. This will provide an important foundation for expanded access to critical mental health programs.

The authors question “national ownership” of MDGs and therefore question their legitimacy. The MDGs are strongly supported throughout the low-income countries, both by civil society as well as by governments, many of whom are developing MDG-based policies. National ownership was vividly displayed in September 2005, when government leaders throughout the developing world protested vociferously and successfully a short-lived attempt of US negotiators to remove the term “Millennium Development Goals” from the UN 2005 World Summit agreement.

Columbia University is involved in the Millennium Village Project, a proof of concept that the MDGs can be achieved in rural Africa by undertaking a holistic approach of integrated interventions in increasing food production, improving access to health care, water, and education, and improving infrastructure. Although the primary focus of health intervention is prevention and treatment of the major killers such as infectious diseases and malnutrition, we are exploring ways to integrate mental health care within the health systems, and we welcome practical suggestions for successful models that merit replication. The approach focuses on community-led development that takes into account social determinants of mental disease, as well as accessible mental health interventions.

The MDGs are a matter of life and death for millions of adults and children. We must do our utmost to ensure their success. The development and public health communities—including mental health professionals—need to work together and not undermine the only shared global development goals we have. Since their adoption in the year 2000, the MDGs have garnered support around the word. If this broad global movement continues to gain momentum and to apply proven solutions to our most pressing problems, the MDGs can be achieved, and with them, so too a significant improvement in mental health around the world.

## References

[pmed-0040056-b001] Miranda JJ, Patel V (2006). Achieving the Millennium Development Goals: Does mental health play a role?. PLoS Med.

[pmed-0040056-b002] Lopez AD, Mathers CD, Ezzati M, Jamison DT, Murray CJL (2006). Global burden of disease and risk factors.

